# Cellular and Molecular Mechanisms of Heart Failure and Sudden Cardiac Death in Hypertrophic Cardiomyopathy and Methods Used for Their Pathogenetic Correction

**DOI:** 10.3390/biomedicines13122926

**Published:** 2025-11-28

**Authors:** Lev Kakturskiy, Yury Belov, Liudmila Mikhaleva, Andrey Lysenko, Zarina Gioeva, Natalia Tikhonova, Nikita Gutyrchik

**Affiliations:** 1 Avtsyn Research Institute of Human Morphology of Federal State Budgetary Scientific Institution “Petrovsky National Research Centre of Surgery”, 117418 Moscow, Russia; levkaktur@mail.ru (L.K.); belovmed@gmail.com (Y.B.); mikhalevalm@yandex.ru (L.M.); dr.lysenkoav@med.ru (A.L.); nb-ti@hotmail.com (N.T.); gyt94@yandex.ru (N.G.); 2 Institute of Medicine, Peoples’ Friendship University of Russia, 117198 Moscow, Russia

**Keywords:** hypertrophic cardiomyopathy, genetic basis, heart failure, atrial fibrillation, sudden cardiac death

## Abstract

**Background/Objectives:** This paper provides a review of the literature data concerning the cellular and molecular mechanisms of heart failure and sudden cardiac death in hypertrophic cardiomyopathy (HCM), and explores approaches used for their pathogenetic correction. **Methods**: This study highlights genetically determined targets of primary damage to the cardiomyocyte ultra-structure—the actomyosin complex of sarcomeres and mitochondria. **Results/Conclusions**: Damage to these structures leads to heart failure and an increased risk of sudden cardiac death, manifesting against a background of phenotypic features such as cardiac remodeling, asymmetric hypertrophy, left ventricular outflow tract obstruction, myofiber disarray, and atrial fibrillation. Both invasive and non-invasive approaches for the pathogenetic management of these fatal complications are characterized.

## 1. Introduction

Hypertrophic cardiomyopathy (HCM) is a genetic disorder characterized by an asymmetrical hypertrophy of the left ventricle (LV), frequently involving the septum, that cannot be explained by secondary causes. HCM can be classified into two sub-categories: obstructive and non-obstructive. It should be noted that in many cases, HCM remains asymptomatic. There are two categories of HCM: obstructive and non-obstructive. In obstructive HCM, the increased mass of the interventricular septum narrows the left ventricular outflow tract (LVOT), leading to decreased LV chamber size with a reduced ejection fraction; excessive cardiac contraction, which can cause mitral regurgitation; atrial dilation; and fibrillation. HCM can involve the right ventricle, though this finding is relatively rare [1,2]. Clinical symptoms of HCM develop due to diastolic dysfunction and myocardial ischemia that cause angina pectoris and cardiac arrythmias. LVOT obstruction can also contribute to HCV manifestations. However, approximately only 5% to 7% of patients with HCM will develop advanced heart failure [3]. HCM is one of the important causes of sudden cardiac death in young individuals [1]. Apical hypertrophic cardiomyopathy (ApHCM) is a variant of HCM which accounts for approximately 10% of HCM cases. It is characterized by hypertrophy predominantly localized in the apical segments of the left ventricle. This cohort of patients generally has a better outcome compared to those with non-ApHCM, showing lower rates of HF and sudden cardiac death (SCD) [4]. As demonstrated by multivariable analysis, age was the only independent predictor of HCM-related mortality, including HF progression, and sudden death events [5].

## 2. The Genetic Basis for HCM

HCM has an autosomal dominant pattern of inheritance, which is linked to mutations in the genes that encode for proteins of the sarcomere’s tropomyosin complex in cardiac muscle cells. HCM is typically caused by an inherited single genetic mutation, which remains unidentified in 40% of HCM patients. More than 11 such mutations have been identified, with the most frequently encountered being mutations in the MYH7, MYBPC3, TNNI3, and TNNT2 genes, which encode the β-myosin heavy-chain, myosin-binding protein C, cardiac troponin I, and cardiac troponin T, respectively [1,6,7]. It has been shown that HCM patients carrying MYH7 R453C most frequently develop progressive HF [8].

HCM exhibits significant variability in its onset and clinical course, ranging from an asymptomatic state (genotype-positive, phenotype-negative, G+/Ph−) to severe manifestations (G+/Ph+) characterized by advanced cardiac remodeling. The latter includes heart hypertrophy, disarrayed myocardial cells, fibrosis, and microvascular disease [9]. In about 30% to 40% of HCM patients with typical clinical signs and morphological characteristics (G–/Ph+), the casual genes remain unknown [6,10].

A study of an international cohort of HCM patients (n = 1468) found that genetic status (genotype-positive vs. genotype-negative) was not a predictor of the clinical course, including HF progression or SCD, in the long term [5]. Recent research suggests that in patients with HCM, all-cause mortality does not differ between genotype-positive (G+) and genotype-negative (G−) individuals, nor does it significantly differ from the expected all-cause mortality in the general U.S. population [11]. However, young HCM patients (20–29 years) had four-fold higher mortality than the general United States population at a similar age [12]. At the same time, there were no significant differences in SCD rates between genotype-positive (G+) and genotype-negative (G−) patients with HCM [13]. In general, an individual HCM patient’s genotype is not a reliable predictor of the disease outcome because the genome is influenced by exogenous factors and epigenetic variability [14]. Future improvements in genetic testing are expected to identify causative gene defects for HCM [1,10]. Researchers have identified FARS2, encoding mitochondrial phenylalanyl-tRNA synthetase, as a potential novel pathogenic gene linked to HCM. Inherited FARS2 deficiency can cause mitochondrial dysfunction, leading to a lack of energy in cardiomyocyte sarcomere and resulting in HF and increased risk of SCD [15].

In rare cases of HCM with no sarcomere gene mutations (G–/Ph+), pathogenic mutations in the *MT-RNR2* gene encoding mitochondrial ribosomal RNA have been identified as a cause [16]. These mutations lead to mitochondrial dysfunction and impaired oxidative phosphorylation and are linked to HF progression.

## 3. Heart Failure

Heart failure (HF) is a clinical syndrome characterized by symptoms and/or signs caused by structural and/or functional cardiac abnormalities that lead to a cardiac output that is insufficient to meet the body’s metabolic demands, resulting in systemic and/or pulmonary congestion [17]. According to SHaRe (Sarcomeric Human Cardiomyopathy Registry) of nearly 7000 patients with HCM, 7.5% developed outflow tract obstruction-related HF over a 15-year period [8]. HF occurs less frequently in patients with non-obstructive HCM than in those with obstructive HCM. This section focuses on the 5% to 7% of HCM (see above) patients who develop advanced HF. Surgical myectomy is highly effective at reversing heart failure symptoms in most cases of obstructive HCM. However, in a small subgroup of patients, myectomy does not prevent the development of HF. Predictors of poor surgical outcomes include significant hypertrophy of the interventricular septum and younger patient age [18]. In some cases, the lack of a positive effect from myectomy is explained by the excessive replacement of contractive myocardial cells by new interstitial fibrotic tissue, which is inherently unable to support the heart’s pumping function [19]. Research confirms that the extent of myocardial fibrosis, particularly in the interventricular septum, is a predictor of HF development [20]. Attention should be paid to interstitial edema as a sign of disease progression [21].

Electron and confocal microscopy of the interventricular septum tissue obtained during myectomy from patients with HCM revealed significant destructive changes in the system of transverse (T)-tubules of cardiomyocytes, which correlated with the severity of HF. These changes manifested as dilation of the T-tubules, their dislocation relative to the Z-lines, a reduction in their density, disruption of their transverse orientation, and, ultimately, complete destruction referred to by the term “detubulation”. T-tubules are important for electrical impulse transmission, and their disruptions lead to electrical instability in the myocardial cells, causing impaired electrical potential and altering the Na^+^ and Ca^2+^ balance [22]. Less than 1% of HCM patients exhibit the so-called primary T-tubule remodeling which stems directly from inherited gene mutations, affecting T-tubule structural proteins such as junctophilin (JPH2), caveolin-3 (CAV3), amphiphysin (BIN-1), etc. [23].

A more detailed analysis of the precise cellular and molecular mechanisms of HF in HCM patients has demonstrated that there is a profound energy deficit in the heart muscle, which is associated with a marked decrease in fatty acid oxidation enzymes, nucleotides, and ATP, as well as an increase in ketone bodies and branched-chain amino acids [24,25].

The K146N mutation in the human β-cardiac myosin heavy-chain gene is a significant factor in the development of HCM which can lead to HF. The mutation slows down the myosin cross-bridge cycle and alters the myosin’s ability to bind and detach from actin filaments [26]. Normally, during diastole, tropomyosin is positioned to block the myosin-binding actin sites, preventing muscle contraction. The initiation of muscle contraction requires calcium ions (Ca^2+^) binding to troponin C. Mutations in troponin or other contractile proteins can increase sarcomere sensitivity to Ca^2+^. This increased sensitivity causes a shift in the troponin–tropomyosin complex, which uncovers the binding actin sites for myosin. It can result in diastolic dysfunction characterized by increased energy consumption, discoordination of the sarcomeres, and impaired relaxation during the diastolic phase. Ultimately, this leads to impaired muscle contraction strength and a reduced ejection fraction, and the body compensates by activating a signaling cascade that causes myocardial hypertrophy.

In HCM, mutations in sarcomere proteins enhance the sensitivity of the myofilaments to calcium ions (Ca^2+^). This increased sensitivity causes the heart muscle to con-tract more forcefully during systole (hypercontractility) and prevents it from fully relaxing during diastole. As more ATP (and oxygen) is required for both systolic and diastolic functions of the heart, the energy load on the myocardium of HCM patients increases dramatically. In turn, this increased energy load leads to an energy deficit reflected in a decreased phosphocreatine/ATP ratio [27]. These processes are considered key factors in the development of HF, even before symptoms appear, e.g., in the preclinical stage.

Myocardial samples obtained during myectomy from HCM patients showed impaired oxidative phosphorylation processes, accumulation of free fatty acids, and severe mitochondrial injuries, including swollen mitochondria and a reduction in the density of their cristae [28]. Using imaging mass spectrometry, researchers studied endomyocardial biopsy specimens from the interventricular septum of HCM patients and assessed the intensity of docosahexaenoic acid (DHA), which is an essential omega-3 polyunsaturated fatty acid [29]. They found that DHA intensity correlated with HF severity. Thus, it is suggested that DHA could be a diagnostic and prognostic marker of HF, as its higher accumulation in severe HF is linked to impaired oxidative phosphorylation of fatty acids and oxidative stress in the hypertrophic heart.

A multicenter, non-interventional, cross-sectional epidemiologic study (TTRACK) conducted in 11 countries has demonstrated that almost 20% of HCM patients had co-existing transthyretin amyloid cardiomyopathy, a condition believed to be the cause of HF in this patient cohort [30].

Interestingly, in about 5% of HCM patients, heart failure develops through a transformation characterized by reduced hypertrophy and chamber dilation. This signifies a distinct remodeling of the clinical and morphological picture toward dilated cardiomyopathy [20].

## 4. Atrial Fibrillation

Atrial fibrillation (AF) is one of the more serious HCM complications. Left atrial dilation due to diastolic dysfunction is a key factor that triggers atrial fibrillation (AF). Diastolic dysfunction creates resistance to blood filling the ventricle from the atrium and causes the left atrium to enlarge. This enlarged atrium provides a larger space where electrical impulses can follow a circular path, a phenomenon known as re-entry. The subsequent atrial fibrosis disrupts the uniform electrical impulse conduction in the heart, exacerbating the re-entry mechanism. A prospective multicenter study of AF patients from the Hokuriku-plus AF Registry revealed that 5.2% of them had HCM [31]. Other studies have demonstrated that AF is frequently associated with HCM, with a reported prevalence ranging from 12.5% to over 25% [32,33]. AF prevalence significantly increases with age in HCM patients, with some studies indicating around 50% prevalence in patients over 70 years old [34]. It was found that AF is more common in HCM patients with mutations of the gene MYH7 [35]. Patients with HCM who develop AF have a higher risk of thromboembolic events. While the exact incidence of thromboembolic events can vary, high plasma levels of B-type natriuretic peptide (BNP) were significantly associated with this complication. Thus, plasma BNP might be a useful biomarker for these adverse clinical events [36]. In patients with HCM, left atrial (LA) remodeling indicated by reduced left atrial reservoir strain (LARS) measured with cardiac magnetic resonance is associated with an increased risk of AF and a poor prognosis for developing HF [37]. A higher atrial fibrosis degree has been observed in HCM patients with persistent AF compared to those with paroxysmal AF [32].

## 5. Sudden Cardiac Death

As the yearly incidence of SCD in adult patients suffering with HCM is <1% [38], the risk of SCD in pediatric patients with HCM is evaluated to be 2.7% [39]. The rates of SCD in HCM patients in Asia were markedly higher than in other regions of the world [40]. HCM is widely considered to be a leading cause of SCD in young athletes [41]. Myocardial ischemia is a major cause of SCD. It can be induced by intramural artery abnormalities, such as intimal hyperplasia and medial hypertrophy, leading to narrowed artery lumens [42]. The authors do not associate these abnormalities with ischemic heart disease. The American College of Cardiology has identified five risk factors for SCD in HCM, including family history of SCD, unexplained syncope, severe left ventricular hypertrophy (over 3 cm), non-sustained ventricular tachycardia, and abnormal blood pressure response during exercise [14]. Other risk factors for SCD in HCM include a high degree of left ventricular outflow tract obstruction, an increase in left atrial diameter, and elevated plasma BNP [38,43]. In young HCM patients, myocardial fibrosis, identified by late gadolinium enhancement on cardiac magnetic resonance imaging, is considered a significant predictor of SCD [44]. Childhood-onset HCM with congestive heart failure is linked to a higher rate of SCD in children than in adults [39].

The subgroup of pediatric HCM patients with the RAS-HCM phenotype (Reduced Aortic Stiffness—hypertrophic cardiomyopathy) is at increased risk of SCD [45]. This phenotype is characterized by an abnormal aortic response to an exercise stress test. Normally, the aorta responds to exercise by relaxing and dilating. In contrast, in patients with the RAS-HCM phenotype, the aorta responds with increased tissue stiffness, which creates significant resistance to the ejection of blood from the left ventricle, provoking ventricular fibrillation and SCD. To identify patients with the RAS-HCM phenotype, diagnostic stress echocardiography is performed. Left ventricular apical aneurysms (LVAAs) are an emerging prognostic factor for SCD in HCM which are observed in approximately 3% of HCM patients. LVAA is diagnosed using contrast echocardiography, where the aneurysm-like bulging of the ventricular wall at the heart apex becomes visible during systole and disappears during diastole [46]. LVAAs can also be detected by cardiac magnetic resonance imaging. From an anatomical point of view, this phenomenon should not be classified as a true aneurysm. It is explained by akinesis of the ischemic areas of the myocardium, which lose their ability to contract and bulge outward during systole. Using PET/CT imaging, researchers explored the correlation between fibroblast activation protein inhibitor (FAPI) and increased 5-year risk of SCD in patients with HCM. All studied subjects with HCM had intense and heterogeneous cardiac FAPI activity in the myocardium. In HCM patients, more segments with FAPI activity were detected than hypertrophic segments [47]. The authors concluded that FAPI uptake was associated with 5-year risk of SCD in patients with HCM. They also demonstrated that in HCM patients, the FAPI amount had a positive relationship with N-terminal probrain natriuretic peptide, high-sensitivity troponin I, and left atrial diameter and a negative relationship with the left ventricular ejection fraction z-score. French scientists discuss potential association be-tween HCM and viral myocarditis. They provided a detailed description of two autopsy cases, where HCM was diagnosed in combination with Herpes Virus Type 6 (HHV6) and/or Parvovirus-B19 (PVB19) in the heart [48]. The widespread introduction of implantable cardioverter–defibrillators (ICDs) has significantly reduced the risk of SCD in patients with HCM [49]. The American College of Cardiology (ACC) has developed a new model for SCD risk stratification for primary prevention in patients with HCM. The ACC algorithm efficiently detects high-risk patients who would benefit from the placement of an implantable cardioverter–defibrillator [50].

## 6. Pathogenesis-Based Treatments of HCM Complications

Septal myectomy is considered the first-choice radical treatment for obstructive HCM to prevent further progression to HF. Surgical myectomy involves direct resection of a portion of the hypertrophied interventricular septum to widen the left ventricular outflow tract. Alcohol septal ablation (ASA) is an alternative option for HCM treatment that involves injecting alcohol into a septal artery, causing localized tissue necrosis and shrinking of the excess heart muscle. Radiofrequency ablation has also been applied for the treatment of obstructive HCM to destroy a small amount of excess heart muscle tissue in the septum using a high-frequency electrical current. After examining the rates of adverse events in patients who underwent septal reduction procedures, researchers pointed out that a low septal myectomy volume was associated with worse outcomes, including higher mortality, longer length of stay, and higher costs [51]. Both septal myectomy and alcohol septal ablation are associated with higher risks of adverse outcomes, such as embolic stroke, ventricular septal defects, and complete heart block [34]. Surgical myectomy effectively relieves LVOT obstruction and its symptoms, but it does not prevent the underlying progression of HCM, as the primary driver of HCM progression is altered cross-bridge behavior caused by these mutations. Failed surgical myectomy can lead to complications such as incomplete resection, ventricular septal perforation, and damage to the heart’s electrical system, including the bundle of His, which can result in atrioventricular block or left bundle branch block. Heart transplants have become an increasingly important option for HCM patients with progressive HF who have received ineffective medical treatment. Compared with heart transplants for other cardiomyopathies, patients with HCM had similar survival rates [52]. Of particular interest are attempts at pathogenetically grounded therapeutic interventions aimed at correcting subtle cellular and molecular abnormalities in HCM. Recent studies show that genotype-negative (G–/Ph+) HCM patients exhibit impaired mitochondrial oxidative phosphorylation and fatty acid oxidation in septal myectomy samples [53]. However, transmission electron microscopy did not reveal significant reduced abundance or fragmentation of mitochondria. At the same time, in genotype-positive (G+/Ph+) HCM patients, no mitochondrial dysfunction was observed, as in this cohort the disease is caused by mutations in sarcomere protein-encoding genes. Thus, the authors suggest that in genotype-negative (G–/Ph+) HCM patients the correction of intracellular mechanisms should be aimed at restoring mitochondrial function. Conversely, in genotype-positive (G+/Ph+) HCM patients, stabilizing sarcomere contractility is the target of therapies. Furthermore, in vitro nicotinamide adenine dinucleotide (NAD+) has been demonstrated to have a beneficial effect on mitochondrial function restoration [53]. Evidence shows that elamipretide, a mitochondria-targeting peptide, acts as an antioxidant and stabilizes cardiolipin in the inner mitochondrial membranes [53,54]. Cardiolipin is a unique phospholipid that is exclusively expressed in the inner mitochondrial membrane, where it plays an important structural role in oxidative phosphorylation. The effects of 1-deoxynojirimycin (1-DNJ) in restoring mitochondria were described in cellular models carrying a mutant mitochondrial gene, MT-RNR2, which is causally implicated in familial HCM [16,55]. 1-DNJ is an iminosugar, a type of sugar analog, in which the oxygen atom is replaced by a nitrogen atom. Iminosugars act as inhibitors of glucosidases and glycosyltransferases, thus stabilizing the structure of intracellular carbohydrates. These research findings, which highlight the restoration of mitochondrial function in the heart, hold promise for the future management of HCM patients.

As regards therapeutic approaches aimed at stabilizing the heart’s sarcomeres, a novel class of drugs, cardiac myosin inhibitors (CMIs), have demonstrated significant benefits, representing a targeted therapeutic advancement for HCM [34]. CMIs work by preventing cardiomyocyte sarcomere hypercontractility, reducing the number of actin–myosin cross-bridges and decreasing ATPase activity in the myosin heavy-chain. Mavacamten, the first cardiac myosin inhibitor, has demonstrated good efficacy in treating HCM [2,56]. Preclinical experiments demonstrated that mavacamten was beneficial for mice with heterozygous human-like myosin heavy-chain mutations. The drug suppressed the development of ventricular hypertrophy, cardiomyocyte disarray, and myocardial fibrosis, and furthermore it attenuated hypertrophic and profibrotic gene expression [57]. Similar results were obtained in preclinical trials of Aficamten, in which a mouse model was used. It suppressed the formation of actin–myosin cross-bridges and decreased ATPase activity in sarcomere myosin [58]. In a systematic review and meta-analysis of 524 HCM patients, Mavacamten was shown to be an effective treatment option for obstructive HCM [59]. Similarly, the clinical trial, involving 41 subjects, has demonstrated the beneficial effects of Aficamten in patients with obstructive HCM [60]. The development of HF in HCM patients taking CMIs can affect the ventricular function, inducing systolic dysfunction [61]. As regards the current management options for HCM, medical therapy using myosin inhibitors is a breakthrough in the management of HCM patients and will emerge as a mainstay treatment option in the future. However, patients should be cautioned about the possible side effects of heart failure due to inhibition of sarcomere contractility. A reduction in actin–myosin cross-bridges can decrease left ventricular ejection fraction (LVEF) and exacerbate heart failure. Therefore, myosin inhibitors are designed to precisely target this mechanism, but their effectiveness is dose dependent. Careful dose titration and regular monitoring are crucial to help balance the beneficial effect of relieving left ventricular outflow tract obstruction with the risk of excessive reduction in the heart’s pumping function.

Modern gene therapy is considered to be a promising approach for HCM that aims to correct the underlying genetic mutations causing the condition. However, this field is still in the early stages of scientific development and is currently limited primarily to preclinical trials [62,63]. In this context, preclinical studies have demonstrated that using an adenine base editor and CRISP technology, it was possible to achieve efficient in vivo genome editing in mice carrying the heterozygous HCM pathogenic variant. Thus, the mutation in the myosin heavy-chain was successfully corrected, preventing the development of hypertrophy, mitigating left ventricular pathological remodeling, and reducing fibrosis of the myocardial tissue [64]. This research area opens prospects for new treatment strategies, including gene replacement, genome editing, allele-specific silencing, and signaling pathway modulation [65]. Though advances in gene therapy for HCM hold great promise, the long-term effectiveness is still uncertain, as some of the “introduced” genes tend to have a short duration of action.

**Figure 1:**
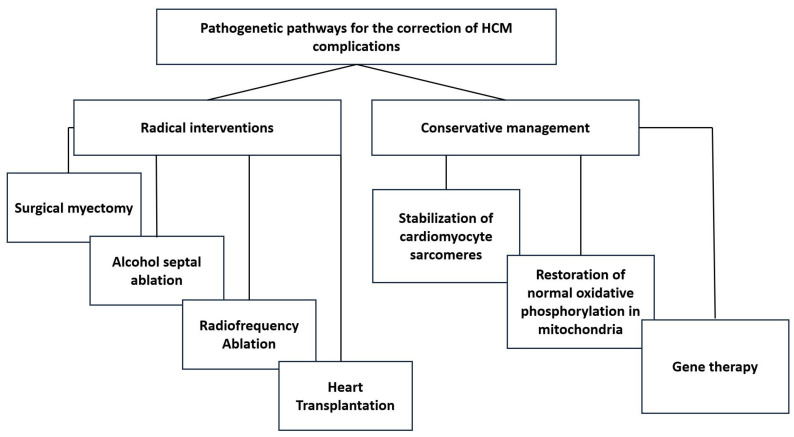
The pathways for the pathogenetic correction of HCM.

The pathways for the pathogenetic correction of HCM complications can be summarized as shown in Figure 1. This implies two main approaches to correction: radical interventions and conservative treatment methods. The details of these pathways are presented below.

## 7. Conclusions

Hypertrophic cardiomyopathy (HCM) is an inherited disease that is frequently caused by pathogenic variants in genes encoding sarcomere proteins. We would like to highlight that the primary molecular cause of HCM development and progression is mutations in genes encoding sarcomere proteins like myosin, actin, myosin-binding protein, and troponins. These mutations lead to the production of sarcomeric cross-bridges that function abnormally, generating excessive force and energy metabolism issues. These primary problems then trigger secondary pathological processes such as inflammation, fibrosis, etc. All other abnormalities are considered secondary. Some HCM patients have mutations in genes that are involved in mitochondrial oxidative phosphorylation. A small subset of patients have the disease without any identifiable genetic mutations. Structural and functional HCM characteristics include heart remodeling with asymmetric left ventricular hypertrophy, primarily involving the interventricular septum, and myofibril dis-array. The most common causes of death in HCM patients include sudden cardiac death and heart failure. Atrial fibrillation with embolic complications also poses a significant threat. Left ventricular outflow tract obstruction is a critical factor in the progression of heart failure in HCM, which often necessitates radical surgical treatment (septal myectomy or heart transplantation). Pathways for the pathogenetic correction of the disease are aimed at normalizing sarcomere structure and oxidative phosphorylation in mitochondria.

## Data Availability

All data and materials are available upon reasonable request. Address to Z.G. (email: gi-oeva_z@mail.ru) or L.K. (email levkaktur@mail.ru), Avtsyn Research Institute of Human Morphology of Federal State Budgetary Scientific Institution “Petrovsky National Research Centre of Surgery,” Moscow, Russia.

## Abbreviations

The following abbreviations are used in this manuscript:

HCM	Hypertrophic cardiomyopathy.
LV	Left ventricle.
LA	Left atrial.
HF	Heart failure.
ApHCM	Apical hypertrophic cardiomyopathy.
SCD	Sudden cardiac death.
G+/Ph−	Genotype-positive, phenotype-negative.
G−/Ph+	Genotype-negative, phenotype-positive.
DHA	Docosahexaenoic acid.
